# Salivary 1,5-Anhydroglucitol and AGEs Are Associated with Postural Instability in Diabetic Foot Patients

**DOI:** 10.3390/medicina61060968

**Published:** 2025-05-23

**Authors:** Lorenzo Brognara, Mar Sempere-Bigorra, Omar Cauli

**Affiliations:** 1 Department of Biomedical and Neuromotor Sciences (DIBINEM), Alma Mater Studiorum University of Bologna, 40100 Bologna, Italy; lorenzo.brognara2@unibo.it; 2 Nursing Department, Faculty of Nursing and Podiatry, University of Valencia, 46010 Valencia, Spain; marsembi@outlook.es; 3 Frailty Research Organized Group, Faculty of Nursing and Podiatry, University of Valencia, 46010 Valencia, Spain

**Keywords:** salivary biomarkers, AGEs, HbA1c, balance, gait, inertial sensors

## Abstract

*Background and Objectives*: Gait and posture alterations are reported in patients with diabetic foot. We evaluated whether gait and postural parameters are associated with a well-known parameter, e.g., glycated hemoglobin levels in blood, and the salivary markers 1,5-anhydro-D-glucitol (1,5-AG) and Advanced Glycation End-Products (AGEs) measured in saliva samples. *Materials and Methods*: Gait and postural impairment was assessed using a wearable inertial sensor, and the evaluation of balance/gait and risk of fall was determined by the Tinetti Scale and Downton Index, respectively. Glycemic control was measured by glycated hemoglobin concentration and fasting glycemia. The salivary concentration of 1,5-AG and AGEs was measured using an enzyme-linked immunosorbent assay. *Results*: Eighty-five patients were evaluated, revealing significant associations (*p* < 0.05) between salivary 1,5-AG and sway path displacement along the medio-lateral axis (rho = 0.365, *p* = 0.017) and sway area (rho = 0.334, *p* = 0.031) during tandem position tests with eyes closed. Salivary AGEs were significantly associated with sway path displacement along the anterior–posterior axis (rho = 0.419, *p* = 0.004) and medio-lateral axis (rho = 0.436, *p* = 0.002) in the tests performed with eyes closed, feet close together, and foam pads, as well as with sway area (rho = 0.387, *p* = 0.007). The concentration of HbA1c was significantly correlated with sway path displacement along the anterior–posterior axis in the tests performed with eyes closed, feet close together, and foam pads (rho = 0.236, *p* = 0.043), as well as with sway area (rho = −0.236, *p* = 0.043). A significant difference was observed in the salivary AGE concentration between patients with previous ulcers versus those without (*p* = 0.035). By applying Bonferroni correction for multiple comparisons, the associations remained significant (*p* < 0.05) for AGE concentration in saliva and postural instability parameters. *Conclusions*: The results suggest a link between salivary glycemic control biomarkers, in particular AGEs and postural changes in patients with diabetic foot, indicating a new interesting filed for further studies on fall risk.

## 1. Introduction

Diabetes mellitus (DM) is a common disease, considered to be a serious public health problem, with a continuously growing prevalence in adults that is expected to rise to 9.9% in 2045 [1]. Among the neurological alterations that diabetes can induce, gait and balance have a great impact. Gait characteristics differ in people with diabetes compared to those without [2,3,4] and affect both people with type 1 and type 2 diabetes [5,6], although most studies have analyzed people with type 2 diabetes due to its high prevalence in the general population. People with diabetes walk more slowly, with shorter steps, a longer stance phase, a wider base of support, greater variability in step time on uneven surfaces, and inadequate pressure distribution across the foot compared to people without diabetes [7,8]. In a longitudinal study, it was observed that people with diabetes had an alteration in gait due to aging earlier than people of the same age without diabetes, with increased stiffness, minimal lifting movement of the feet off the ground, body bradykinesia associated with gait, and less postural stability [9]. Other studies have shown that people with diabetes have less overall mobility and strength in the ankles during walking compared to people without diabetes [10,11,12,13,14]. Recently, increased flexion/extension and lateral movement during linear gait and turns, assessed using accelerometry, were found at major joints (ankles, knees, hips, shoulders, and neck) in people with diabetes. This increased movement or movement error was partly the result of tremors in the 8 and 16 Hz bands, suggesting that part of this increased joint motion is likely of central origin and not solely related to motor and sensory loss in the lower extremities [15]. The sway along the anteroposterior axis during gait correlates significantly with poor glycemic control, i.e., higher glycated hemoglobin concentration, more severe neuropathy, increased risk of falls, increased muscle weakness, functional limitation at the ankle, and the presence of diabetic foot ulcers. [16]. Alterations in posture and gait increase the risk of falls, increasing the costs, morbidity, and mortality associated with diabetes [17,18,19,20]. The increase in postural instability found in individuals with type II diabetes compared to controls has been associated with increased number of falls [21]. Several factors are thought to influence the impairment of gait and postural stability in people with diabetes such as sensory impairment (presence of neuropathic ulcers, impaired vibration, and protective sensation) [22,23,24,25], alterations of the vestibular system [26,27,28], decreased strength in the lower limbs (force production capacity) [29,30], and impairment of the central nervous system [31,32,33]. Wearable inertial sensors (IMUs) use accelerometers, gyroscopes, and magnetometers to provide very accurate estimates of gait and postural parameters in patients with diabetes [34,35,36]. Blood HbA1c concentration, recommended as a standard of care for testing and monitoring diabetes control, is usually elevated by episodes of glycemic instability with late sequelae, which include foot ulcerations [37,38]. However, new biomarkers of peripheral nerve disease in diabetes have a potential role in diagnosis, clinical management, and research [39,40,41]. Among the three biomarkers, 1,5-anhydro-D-glucitol (1,5-AG) is a monosaccharide, coming mainly from food, that is very similar to glucose in its structure [42,43]. The measurement of plasma levels of 1,5-AG offers a relatively new option for assessing blood glucose metabolism. In the normoglycemic context, 1,5-AG remains at high but constant concentrations in the blood [43]. 1,5-AG is freely filtered by the glomeruli and reabsorbed in the renal tubule, with a small amount excreted, corresponding to dietary intake [44]. However, in the context of poor diabetes control and, in turn, hyperglycemia, when glucose levels rise above the renal threshold for glycosuria (≥10 mmol/L), the elevated amounts of glucose block tubular reabsorption of 1,5-AG, causing a decline in serum levels of 1,5-AG [43]. 1,5-AG is inversely associated with an increase in postprandial glucose concentrations and high HbA1c concentrations, suggesting this biomarker as a marker of glucose variations that occur mainly in the postprandial period. [42,45], reflecting both fasting and post-prandial glycemic response [46]. The appeal of 1,5-AG for use in the treatment of diabetes is due to the possibility that it may capture additional information about glycemic excursions that is not reflected in HbA1c values, the most common marker of diabetes control. Furthermore, measurement of 1,5-AG does not require fasting, and the colorimetric assay has stable reagents and is easily automatable [47]. 1,5-AG has been proposed to the FDA (Food and Drug Administration, federal agency of the United States Department of Health and Human Services) for consideration as an index of glycemic control in diabetic patients. Interestingly, the concentration of 1,5-AG in saliva is closely correlated with 1,5-AG levels in blood and inversely correlated with blood glucose and glycosylated hemoglobin levels. In addition, this biomarker measured in saliva seems to be robust across different ethnicities, irrespective of body mass index, age, and gender [48]. Another biomarker associated with poor glycemic control is the presence of Advanced Glycation End-Products (AGEs) [49,50]. This is a complex and heterogeneous group of compounds, formed via a non-enzymatic reaction between reducing sugars and amine residues on proteins, lipids, or nucleic acids, that have been implicated in diabetes complications [51,52,53]. AGEs are also present also in saliva samples of diabetic patients [51,54,55].

AGE-induced alterations of functional and mechanical properties of tissue via crosslinking intracellular and extracellular proteins and on myelin can elicit immunological responses that may lead to demyelination [56,57,58,59,60,61]. A recent study reported that low plasma 1,5-AG is closely associated with impaired peripheral nerve function and peripheral neuropathy in type 2 diabetes [62]. Interestingly, the reduction in the amplitude of nerve action potentials and in the conduction velocity in lower limbs remained correlated with plasma 1,5-AG concentration even after controlling for the effects of glucose reduction afforded by drugs [62]. Regarding AGEs and their relationship with diabetes-induced neurological alterations, a previous study reported that the accumulation of AGEs measured by skin autofluorescence was associated with a higher risk of signs of neuropathy years later, and the highest quartile of skin AGE levels was associated with the worst neurological test results in patients with type 1 diabetes [63]. A high peripheral AGE level is associated with greater cognitive decline in older adults with and without diabetes [64]. Few studies, however, have suggested that these biomarkers could be potentially associated with gait and posture alterations in patients with diabetic foot. We hypothesize that higher levels of 1,5-AG and AGEs in saliva are related to worse gait and posture performance in patients with diabetic foot. Specifically, they are related to alterations observed in other studies using inertial sensors in people with diabetes, such as a reduction in walking speed and step length, an increase in the double support time of feet on the ground, and an increase in step time. As a comparison, the concentration of a widespread marker of glycemic control, e.g., Hb1Ac concentration in blood, was also assessed with gait and posture parameters. To our knowledge, this is the first study to compare the use of innovative diagnostic tools, including metabolites in salivary samples and the measurement of gait and postural data with wearable inertial sensors.

## 2. Material and Methods

### 2.1. Study Design

An observational study was conducted on consecutive diabetic foot patients attending podiatric appointments in a healthcare center located in the province of Valencia (Spain). This was a case–control study of diabetic and non-diabetic patients conducted on institutionalized individuals to explore the relationships between cognitive functions, symptoms of depression, and peripheral sensory neuropathy. The data were collected by a medical doctor, a psychologist, and a group of nurses and podiatrists. The evaluations were performed in three nursing homes located in the province of Valencia between January 2021 and January 2022. This work was completed with the approval of the University of Valencia Ethics Committee for Human Research (Reference: H20190330195344), and all the procedures were undertaken in accordance with the ethical requirements of the Helsinki Declaration.

### 2.2. Gait and Posture Evaluation

**Figure 1:**
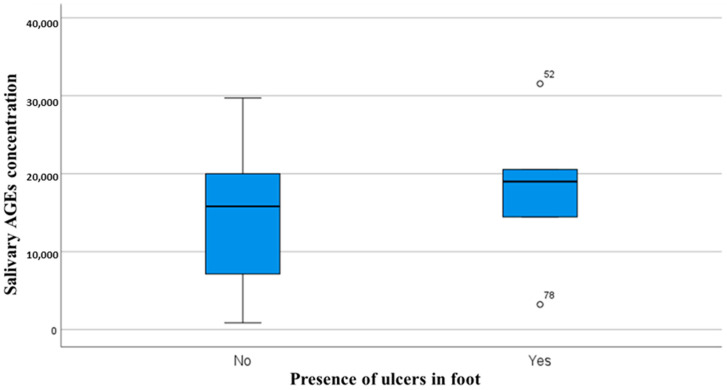
Relationship between salivary AGE levels and presence or absence of diabetic foot ulcers.

A complete gait and postural analysis was performed using two validated Inertial Measurement Units (IMUs): mSway by mHealth Technologies^®^ (Bologna, Italy) and Wiva Science inertial sensors by Letsense^®^ (Bologna, Italy) [16,65]. The system used to analyze gait parameters was Wiva Science, a validated wearable inertial system (ICC > 0.81) that contained a sensor (inertial measurement unit [IMU]) [66]. This sensor consists of a tri-axial accelerometer, a tri-axial gyroscope, and a magnetometer for detecting several spatiotemporal parameters, as well as a micro-electro-mechanical system (MEMS) designed to capture motion and translate mechanical energy into electrical energy by applying an algorithm [67]. The sensor was placed in the lumbar area on the L5 spinal segment using an elastic band, according to the manufacturer’s recommendations. The gait assessment was conducted using the 10-Meter Walk Test, a widely validated tool for detecting gait impairment [68,69,70]. Patients were instructed to walk at their usual speed for a distance of 10 m. Upon reaching the halfway point, they were required to pause for 3 s while standing still. After this brief pause, they turned around, waited for another 3 s, and then began walking back towards the starting point. After this procedure, the Wiva Science sensor sent the information collected to a computer via Bluetooth, and the data were stored in the “The Biomech Studio software” (Version 1.6.1.14687, LetSense Group srl., Bologna, Italy). Many other studies have been carried out using these sensors with similar methodologies for studying gait disturbances in different pathologies and disorders [71,72,73,74]. The following parameters were thereby obtained: speed, cadence, stride length, stance duration, swing duration, step length, and double and single support duration. The mSway system by mHealth Technologies^®^ (mHealth Technologies, Bologna, Italy) was used to analyze postural parameters. It features a tri-axial accelerometer, gyroscope, and magnetometer that measure sway during six 30 s sessions, including eyes open/closed, with or without foam pads (4 cm thick, 35 kg/m^3^), and in both feet-together and tandem positions [75,76]. The sensor was placed at the L5 lumbar level using an elastic band (see Figure 1). Patients maintained the positions independently, with practitioners present for safety, especially during tests with closed eyes and feet together. The gait and postural assessments were carefully randomized and then administered to the patients to ensure a fair evaluation of their mobility and stability. The mSway device transmits the collected data to a computer via Bluetooth for storage and analysis. The following parameters of gait and posture were evaluated: speed [m/min], ratio between the length and duration of the stride, cadence [steps/min], stride length [cm], stance duration [s], step length [m], stance duration [% of gait cycle], swing duration [% of gait cycle], single and double support duration [%], sway area [mm^2^/s], sway path anteroposterior [mm/s], and sway path mediolateral [mm/s].

### 2.3. Analysis of Biomarkers

The saliva samples were collected using the Salivette^®^ system ((Sarstedt, Nümbrecht, Germany), following the manufacturer’s recommendations. Each donor was first asked to rinse their mouth with water. They were then asked to chew the Salivette roll for 2 min so that it could absorb their saliva. The chewed Salivette roll was placed into the Salivette^®^ tube, which was centrifuged at 2500× *g* for 10 min at 4 °C. After centrifugation, the supernatant was transferred into a collection tube. The individual samples in separate collection tubes were also frozen at −80 °C until analysis.

### 2.4. AGEs and 1,5-AG Analysis

Salivary concentrations of AGEs were determined in duplicates using the Advanced Glycation End-Products (AGEs) Assay Kit (abcam, Cambridge, UK, reference Ab273298) and 1,5-Anhydroglucitol (1,5-AG) Assay Kit (colorimetric) (abcam) Ab284531 in accordance with the manufacturer’s instructions. https://www.abcam.com/en-us?srsltid=AfmBOopd7F1wQkYubBEKyai0hTcj0TEh27RSosvUCPsPSqZeP4JQQiYM (accessed on 2 April 2025).

The concentrations of AGEs were calculated using the standard curve of AGE–bovine serum albumin.

### 2.5. Blood Glycated Hemoglobin

After 12 h of fasting, blood samples were taken by puncture of an antecubital vein. The sample was divided into two parts: one was preserved in EDTA in a BD Vacutainer tube (Stockholm, Sweden, Ref 367525), and the HbA1c concentration was measured using high-performance liquid chromatography.

### 2.6. Statistical Analysis

Descriptive statistics, including measurements of the central tendency (mean), standard error of the mean (SEM), and range values were used to describe all the quantitative variables. The normal distribution of each variable was estimated with the Kolmogorov–Smirnov test. The Mann–Whitney U and Kruskal–Wallis tests were used to compare means between the quantitative variables, and the Chi-square test was used to compare proportions. Spearman’s linear correlation coefficient was used to establish the correlation between the quantitative variable tests. Bonferroni correction was also used to correct for multiple testing to minimize the risk of type I errors in these association analyses. Statistical significance was set at *p* < 0.05. Statistical analysis was performed using the SPSS 28.0 software package (SPSS Inc., Chicago, IL, USA).

## 3. Results

### 3.1. Sample Characteristics

The sample consisted of 85 patients, 46 males and 39 females. A total of 18 patients (21.2%) had a diagnosis of type 1 diabetes, and 67 patients (78.8%) had a diagnosis of type 2. The participants had a mean age ± SEM of 68.1 ± 1.3 (range, 20–87). As for the body mass index (BMI), 26 patients (30.6%) had a normal weight, 39 patients (45.9%) were overweight, and 20 patients (23.5%) were obese. The patients’ mean value of HbA1C (mmol/mol) was 52.9 ± 1.3. All the patients (aged 18 years or older) with a diagnosis of diabetes (type 1 or type 2) were included in this study. Patients with cognitive impairment, active ulcers on the feet, lower limb injuries or fractures in the previous 6 months, retinopathy, or conditions causing major gait and posture disorders such as amputation were excluded.

### 3.2. Correlation Analyses

Table 1 shows the correlation coefficients and p values between the salivary concentration of the two biomarkers and gait and postural variables assessed using the inertial sensors. HbA1c concentration was significantly associated with salivary AGE concentration (rho = 0.394, *p* = 0.009), whereas no significant correlation was found between Hb1Ac and salivary 1,5-AG concentration (rh = −0.203, *p* = 0.221) (Table 1) or between salivary AGEs and 1,5-AG (*p* = 0.452). No significant associations were found between fasting glycemia and the concentration of the two biomarkers in saliva (*p* = 0.108 for 1,5-AG and *p* = 0.141 for AGE concentration).

The most significant correlations were found between the salivary 1,5-anhydroglucitol concentration and the sway path of displacement along the medio-lateral axis, recorded during tests performed with eyes closed and feet in the tandem position (*p* = 0.017), and sway area, recorded during tests performed with eyes closed and feet in the tandem position (*p* = 0.031), whereas the salivary AGE concentration was significantly associated with some postural parameters such as the sway path of the displacement along the antero-posterior axis, recorded during tests performed with eyes closed and feet close together (*p* = 0.004), the sway path of the displacement along the medio-lateral axis, recorded during tests performed with eyes closed and feet close together (*p* = 0.002), and sway area, recorded during tests performed with eyes closed and feet close together (*p* = 0.007). The concentration of HbA1c was significantly correlated with the tandem sway path along the antero-posterior axis (rho = 0.236, *p* = 0.043), the sway path along the antero-posterior axis performed with feet close together (rho = 0.246, *p* = 0.034), and the tandem sway area (rho = −0.236, *p* = 0.043). By applying Bonferroni correction for multiple comparisons, the associations remained significant (*p* < 0.05) for the AGE concentration in saliva and postural instability parameters. In contrast, the significance of associations between Hb1AC and 1,5AG and postural instability parameters was lost.

A significant difference in salivary AGE concentration between patients with previous ulcers versus those without was observed (*p* = *0*.035) (Figure 1), whereas insignificant differences were observed for Hb1Ac (*p* = 0.44) or 1,5-AG (*p* = 0.38) in patients with or without ulcers.

**Figure 2:**
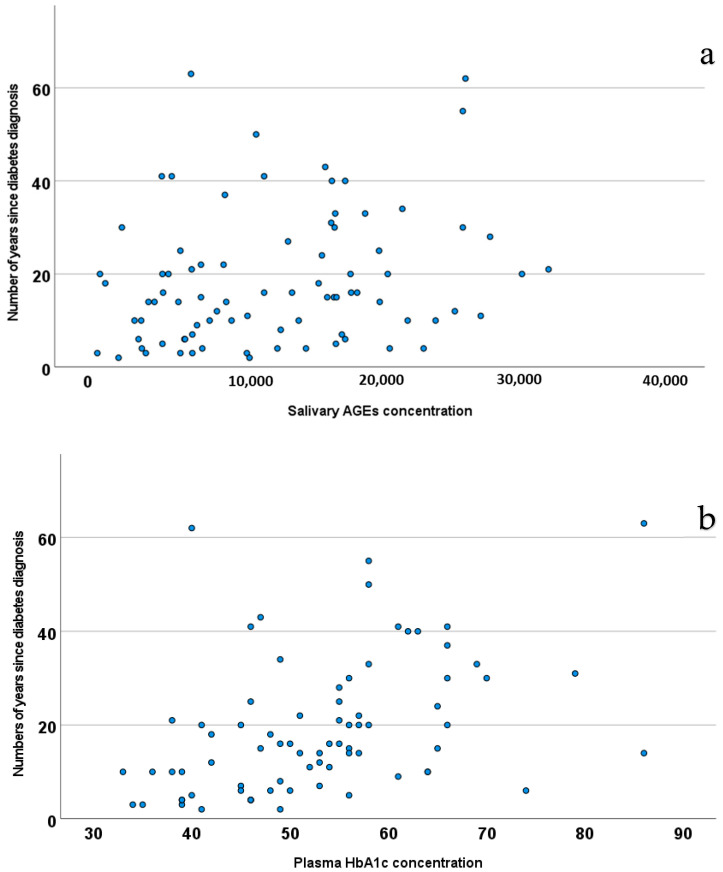
Correlation analysis between salivary AGEs (**a**), plasma Hb1Ac (**b**) concentration, and years passed since diabetes diagnosis (number of years since diagnosis of diabetes).

Finally, analyzing the total sample again, a statistically significant positive relationship was found between AGEs and the number of years living with the diagnosis of diabetes (*p* = 0.030) (Figure 2): as the salivary AGE concentration increases, on average, the duration of living with a diabetes diagnosis lengthens (rho = 0.253, *p* = 0.03), in a similar manner to the Hb1Ac concentration in plasma (rho = 0.495, *p* < 0.001).

## 4. Discussion

Several recent studies have focused on the multifactorial analysis of diabetic foot complications and on the pathophysiological relevance of the AGE concentration in diabetes [77]. Our findings confirm that AGEs can be considered effective biomarkers for early diagnosis of diabetes complications such as ulcers [78]. Glycation is an important mechanism in the development of diabetic complications, and AGE formation is susceptible as a result of protein fragmentation and oxidation of lipids derived from HbAIC variability. The findings of our study showed, for the first time, a relationship between AGEs measured in saliva samples and diabetic foot complications, and are consistent with those of Vouillarmet and co-workers, who reported that the AGE concentration in skin, measured through autofluorescence, is associated with neuropathic foot complications in diabetes [79]. As for clinical variables, previous history of foot ulcers was closely associated with high AGE levels [79] and high Hb1Ac measured in blood samples, at least for type 1 diabetes patients [80,81]. An important process related to several pathophysiological mechanisms and glycation with AGE levels is certainly involved in impaired wound healing [82]. This result is very significant for clinical practice in order to better identify patients at risk of ulcers and complications during tissue damage repair processes.

Furthermore, we found a statistically significant correlation between AGEs and the sway path of displacement along the medio-lateral axis recorded during tests performed with eyes closed (OC_SP_ML Axis). The length of the sway path is an important measure for a quantitative assessment of postural control, and previous studies have shown a relationship between poorer cognitive performance in women, poorer glycemic control, diabetic sensory neuropathy, and sway path [16,83]. Wearable-based postural analysis certainly is an interesting field for future research and may open new horizons in the prevention of the development of diabetic complications.

The results of this study are consistent with our other studies and confirm the relationship between the sway path (the postural variable measured using a wearable sensor), diabetic neuropathy tests, and AGEs [16,65]. The sway path is the length of oscillation of the Center of Mass (CoM) during oscillation on the anterior–posterior axis or the medio-lateral axis. We observed that this parameter is more closely correlated than others with the complications of diabetic foot and could represent a biomarker for identifying patients at risk, and as such, future research should focus on this parameter more than others. A previous study demonstrated that impaired postural control during the quiet stance observed in type 1 and 2 diabetes correlated well with a higher prevalence of falls [84].

On the other hand, no correlations were found between salivary 1,5-AG and AGE concentrations and gait parameters. Although gait and postural alterations interact with each other, their pathophysiology alterations do not overlap [7,85]. Our results showed correlations between an increased salivary AGE or 1,5-AG concentration and posture parameters, although these associations were not the same for each posture parameter and depended on the type of biomarker. The different correlations between variables must be considered from the perspective of diversity between biomarkers. Compared to blood HbA1C and the salivary AGE concentration (which were correlated with each other), 1,5_AG is in fact more closely associated with glucose fluctuations and postprandial glucose metabolism. It has been demonstrated that microvascular and macrovascular complications are not only related to fasting and long-term hyperglycemia, but also to glycemic oscillations, such as postprandial hyperglycemic peaks [79,82]. Glycated hemoglobin (HbA1C), the gold standard test in glycemic monitoring, representing a marker of hyperglycemia in the 2–3 months preceding the test, does not provide information on short-term glycemia and glycemic fluctuations. Furthermore, considering the high pre-analytical variability of this test, possible alternative markers and those to be used alongside HbA1C in blood sugar monitoring have been studied for some years: these include 1,5-anhydroglucitol, which with a single test is capable of providing a specific measure of hyperglycemia in the 2 weeks prior to sampling [78,79,82]. The association between the postural parameters measured with the wearable sensor and the AGEs and 1,5-AG measured in saliva is an important asset for future studies, as it could be a useful biomarker in studies of postural stability and, thus, in evaluating the risk of falls.

The ongoing evolution of diabetes foot care is strongly related to a series of innovative health technologies. It is important to investigate these tools in the field of diabetic foot care, which could have multiple potential implications, including the identification of new variables that are closely associated with the progression of specific diabetes-induced complications. Because of their ability to produce a large and heterogeneous amount of data, wearable sensors and salivary swabs could become a very useful technical tool to help practitioners, ensuring more precise screening for patients with diabetic foot complications, and deserve further investigations.

Balance impairments and the subsequent increased risk of falls in older adults with type 2 diabetes have been reported [86,87]. The ability to maintain balance is known to be a complex skill that requires a combination of multiple sensorimotor and cognitive processes. Emerging evidence suggests that diabetes-related subtle declines in sensory functions (somatosensory, visual, and vestibular), metabolic muscle function, and executive functions may also contribute to the increased risk of falls in older adults with type 2 diabetes [78,88]. In our study, we observed a significant correlation between HbA1c levels and various variables that reflect postural control and balance (sway path and sway area). Importantly, we still confirmed the presence of significant, though less powerful, associations (*p* < 0.05) between salivary AGE concentration and postural instability parameters, not observed for blood HbA1c and salivary 1,5 AG concentration, suggesting that among the three biomarkers, AGEs appear to be promising for helping to evaluate diabetic patients at high risk of postural instability and falls. Future studies are needed to compare salivary and blood AGEs in order to assess their sensitivity and specificity regarding stratification of patients for falls risk.

The increase in instability and risk of falling underscores the need to monitor biomarkers measured in biological fluids, such as saliva, alongside postural parameters. Specifically, measures such as anterior–posterior sway can help differentiate between older adults who are at risk of falling [89].

Our study also has some limitations that should be taken into account for future studies. First, patients did not receive a vascular impairment examination (such as the ankle brachial index) to confirm the presence of diabetic angiopathy, and as such, further research should be conducted to validate our results, since neurological complications in diabetes could be either due to poor glycemic control and other comorbidities, especially in type 2 diabetes [90,91,92]. The association between neuropathy and posture-related parameters was not assessed, and we cannot infer the mechanisms producing impairment in posture changes. Although peripheral sensory neuropathy seems to be the primary factor in postural abnormalities in diabetic patients [93,94], the scientific evidence available does not rule out diabetes per se, and other neuropathies (central, autonomic, and motor) or an inability to fully exploit optical and inertial information about posture [93,95] also play a role. Impairment of neuromuscular function could also be involved, since the mechanisms related to muscle activity deviations are present in diabetic subjects, although these are not directly related to neuropathy [96]. The role of visual and vestibular impairments in posture abnormalities is an aspect that clearly warrants future studies, in view of the fact that AGE and 1,5-AG levels also correlate with diabetic retinopathy [97,98,99]. We must recognize that the limited size of this study is the main limitation to generalizing the present findings, but we believe that this pilot study is a necessary preliminary step to explore novel findings, such as the association between salivary biomarkers related to glycemic control and postural impairment. The most significant associations between postural instability parameters and the analyzed biomarkers were found for salivary AGE concentration. However, it should also be kept in mind that AGEs may be involved in pathogenesis not only of diabetes but also of other metabolic and cardiovascular diseases, chronic degenerative diseases, neurological diseases, and cancers, and it has been suggested as a biomarker of oxidative stress. The lack of a control group prevented us from assessing whether these biomarkers measured in saliva are also useful for evaluating postural instability in non-diabetic older individuals, which was beyond the scope of this study, focused on gait and posture parameters of diabetic foot patients at high risk of falls due to these neurological alterations.

The results obtained with 85 diabetic patients in the present study may guide future research in this emerging field of research, and a comparison between type 1 and type 2 diabetes needs to be performed in order to study if there any differences. In addition, patients selected were all institutionalized in nursing homes, which may not represent the broader diabetic population and limits generalization. Future adequately powered randomized clinical trials, incorporating vascular and neuropathic evaluations or the effects of other comorbidities such as retinopathy, are needed to explore the relationship between salivary biomarkers and gait and postural parameters. Testing will be limited to certain parameters due to sensor constraints, but longer wearing times or different placements of inertial sensors could yield stronger results in future studies.

## 5. Conclusions

Our study suggests two novel biomarkers measured in saliva as suitable tools for investigating the relationship between glucose metabolism in diabetes and posture alterations. Salivary AGE concentration, in particular, may offer a low-cost tool for identifying individuals at risk of falls and ulcer recurrence in diabetic foot care. Longitudinal studies are required to validate its sensitivity and specificity compared to HB1Ac and other biomarkers related to these complications.

## Figures and Tables

**Table 1:** Correlation coefficients and *p* values between the salivary concentration of the two biomarkers and gait and postural variables assessed using inertial sensors.

	Salivary 1,5-Anhydroglucitol Concentration		Salivary AGE Concentration	
	Correlation Coefficients	*p* Values	Correlation Coefficients	*p* Values
Age	0.074	0.635	0.106	0.472
Body mass index (BMI)	−0.012	0.937	−0.129	0.383
Glycemia (fasting glucose concentration in plasma)	−0.269	0.108	0.234	0.141
HbA1c (glycated hemoglobin concentration in plasma)	−0.203	0.221	0.394	**0.009**
Speed	0.217	0.173	0.207	0.168
Cadence	−0.073	0.651	−0.033	0.83
Stride_Length_centimeters	0.278	0.078	0.13	0.389
Stance_Duration	−0.046	0.773	0.042	0.781
Swing_Duration	−0.024	0.884	0.102	0.998
Double_Support_duration	−0.022	0.89	0.056	0.713
Single_Support_Duration	−0.024	0.884	0.102	0.998
mStep_Length_LR_cm	0.227	0.153	0.161	0.286
mStep_Duration_LR_ms	0.047	0.772	0.048	0.752
mStep_Duration_LR_s	0.052	0.748	0.042	0.784
mStance_Duration_LR	−0.071	0.659	0.047	0.755
mSwing_Duration_LR	0.011	0.946	−0.004	0.978
EO_SP_AP_axis	−0.253	0.106	−0.136	0.36
EO_SP_ML_axis	−0.099	0.534	−0.114	0.444
EO_Sway_Area	−0.077	0.629	−0.194	0.191
EO_Foam_SP_AP_axis	−0.196	0.214	−0.209	0.159
EO_Foam_SP_ML_axis	−0.251	0.109	−0.031	0.834
EO_Foam_Sway_Area	−0.173	0.273	−0.119	0.424
EO_Tandem_SP_AP_axis	−0.163	0.301	0.108	0.47
EO_Tandem_SP_ML_axis	−0.11	0.488	0.056	0.706
EO_Tandem_Sway_Area	−0.031	0.848	0.086	0.565
EO_TF_SP_AP_axis	−0.087	0.585	0.143	0.337
EO_TF_SP_ML_axis	−0.097	0.542	0.133	0.371
EO_TF_Sway_Area	−0.073	0.648	0.106	0.479
EC_SP_AP_axis	−0.071	0.653	−0.244	0.099
EC_SP_ML_axis	−0.132	0.406	−0.04	0.79
EC_Sway_Area	−0.069	0.664	−0.104	0.486
EC_Foam_SP_AP_axis	−0.013	0.935	−0.13	0.382
EC_Foam_SP_ML_axis	0.067	0.671	−0.022	0.883
EC_Foam_Sway_Area	0.015	0.926	−0.068	0.649
EC_Tandem_SP_AP_axis	0.217	0.167	−0.057	0.705
EC_Tandem_SP_ML_axis	0.365	**0.017**	0.052	0.728
EC_Tandem_Sway_Area	0.334	**0.031**	−0.107	0.476
EC_TF_SP_AP_axis	0.182	0.256	0.419	**0.004**
EC_TF_SP_ML_axis	0.209	0.183	0.436	**0.002**
EC_TF_Sway_Area	0.192	0.224	0.387	**0.007**

Abbreviations: EC: test performed with eyes closed; TF: test performed with feet close together; ML: medio-lateral; AP: antero-posterior; SP: sway path. Values in bold indicated significant correlations.

## Data Availability

Data are contained within the article.
